# INTEREST GROUP SESSION—ALZHEIMER'S DISEASE AND RELATED DIMENTIAS: OPPORTUNITIES AND CHALLENGES TO DEVELOPING AND TESTING PRAGMATIC ADRD INTERVENTIONS

**DOI:** 10.1093/geroni/igz038.1324

**Published:** 2019-11-08

**Authors:** Abraham A Brody, Laura N Gitlin

**Affiliations:** 1 NYU Rory Meyers College of Nursing, New York, United States; 2 Drexel University, Philadephia, Pennsylvania, United States

## Abstract

Many clinical trials have been performed to develop the evidence for caring for persons with Alzheimer’s Disease and Related Disorders (ADRD) in tightly controlled settings. These trials have found efficacy of a wide spectrum of interventions to address issues from advanced care planning to behavioral and psychological symptoms of dementia (BPSD). However, few ADRD interventions have been tested in wide-scale pragmatic fashion in long term supportive settings (LTSS) such as nursing homes, primary care clinics, hospices, or community based organizations. This is due to a variety factors, principle amongst them are the difficulty in implementing pragmatic trials, and that many of the interventions developed in tightly controlled settings are not directly translatable to real-world settings. Without translating and testing interventions in real world settings, the evidence base remains largely inaccessible to the end user, the persons with ADRD and their caregivers. Moreover, effectiveness remains unclear. The lack of pragmatic trials in ADRD exists despite significant recent investment from the NIH Office of the Director in a health systems collaboratory to support pragmatic clinical trials. In 2018, NIA therefore released a call for 2-phase intervention development and pragmatic trial testing via an R61-R33 mechanism (PAR-18-585). Four proposals were funded in September 2018 from this PAR. This symposium will explore the opportunities and challenges present in developing and testing pragmatic interventions in ADRD in LTSS. The speakers will also share specific scientific methodological and implementation questions that need to be addressed in applying for pragmatic trial awards.

